# The Durability of Intravenous Hydroxocobalamin in Vasoplegia

**DOI:** 10.7759/cureus.38307

**Published:** 2023-04-29

**Authors:** Madeline Blaha, Meghan Blais, Logan Olson

**Affiliations:** 1 Department of Pharmacy, Nebraska Medicine, Omaha, USA

**Keywords:** hemodynamics, distributive shock, b12, hydroxocobalamin, vasoplegia

## Abstract

Objectives

Refractory distributive shock (vasoplegia) has been treated with intravenous (IV) hydroxocobalamin (B12), but its use is poorly characterized. The objective of this study was to quantify the duration of hemodynamic improvement after B12 administration.

Materials and methods

This was a retrospective chart review of adult patients who received IV B12 while on vasopressors in the intensive care unit. Patients were divided into two groups: responders (≥10% decrease in baseline vasopressor requirements within 60 minutes of B12 administration) and non-responders.

Results

A total of 16 patients were included, and five (31%) met the ‘responder’ criteria. The median time to respond was 15 minutes, and the response was maintained for a median of 210 minutes. The baseline median norepinephrine equivalent (NEE) rate was 32.9 mcg/min in responders and 24.7 mcg/min in non-responders. Responders’ NEE requirements decreased to 16.7 mcg/min after 15 minutes and 14.8 mcg/min after 60 minutes. All responders and 10 (91%) non-responders were mechanically ventilated; both groups were mostly male (60% and 91%) and had a median age of 54 years and 58 years, respectively. A total of 4 (80%) responders and 10 (91%) non-responders died while hospitalized. IV B12 was administered as 5 g over 15 minutes in all but two patients (one responder and one non-responder), who each received 5 g of B12 over 360 minutes.

Conclusion

Vasopressor requirements decreased rapidly in 31% of patients after B12 administration and remained so for a median of 210 minutes.

## Introduction

Vasoplegia can result from multiple etiologies, including cardiopulmonary bypass, sepsis, and a liver transplant [1-18]. It is characterized by low systemic vascular resistance leading to significant hypotension despite normal or high cardiac output, euvolemia, and vasopressors [1-4,19-22]. This refractory state may, in part, be due to dysregulation of nitric oxide (NO) release, production, or signaling, resulting in uncontrolled vascular smooth muscle relaxation and vasodilation [3,5-7].​ Intravenous (IV) hydroxocobalamin (B12) represents an emerging therapeutic option for vasoplegia, given its inhibition of inducible nitric oxide synthase and ability to scavenge NO directly [1,3,6,7].

Previous literature has focused primarily on the use of B12 during or after cardiopulmonary bypass [5-15,18], but a few case reports/series have also described its use in the setting of septic shock or liver transplantation [1-4,16,17]. Limited reports provide any evaluation of B12’s duration of effect, and those that do report an effect persisting beyond the observation window or complete resolution of vasopressor requirements, with only one non-cardiac surgery patient evaluated throughout these case reports [1,7-9,14]. One cardiac surgery study described the termination of the effect on hemodynamic values at approximately 3.5 hours, but vasopressor requirements remained lower than previous for the duration of the observation period [8]. None of the aforementioned reports regarding duration of effect attempted to delineate between those that do or do not respond to B12. The objective of this study was to determine the duration of the effect on vasopressor requirements in all patients that respond to IV B12 in the Intensive Care Unit (ICU). 

## Materials and methods

Study design

This was a retrospective chart review of adult patients (≥ 19 years old) who received IV B12 while on vasopressors in the ICU at Nebraska Medicine between June 1st, 2018, and June 30th, 2021. If more than one B12 dose was administered during hospitalization, only the first dose was evaluated. Individuals were excluded if they were pregnant or legally confined. The study was approved by the local Institutional Review Board, and consent was not required for participation. 

The primary outcome was to quantify the duration of IV B12’s effect on vasopressor requirements in those that responded. Vasopressor requirements were recorded in norepinephrine-equivalent (NEE) rates every 15 minutes for 12 hours after B12 administration. NEE rates were calculated using a previously published formula: (Norepinephrine, mcg/kg/min) + (Epinephrine, mcg/kg/min) + (Dopamine, mcg/kg/min/150) + (Phenylephrine, mcg/kg/min/ 10) + (Vasopressin, units/min/0.4) [18,23]. Patients were classified as “responders” if they experienced a ≥10% decrease in baseline vasopressor requirements within 60 minutes of the B12 dose [10]. B12 activity duration was defined as the time from administration to the first increase in NEE requirements. 

Secondary outcomes were hospital length of stay (LOS), ICU LOS, and in-hospital mortality. Baseline demographic data was collected to characterize the population, which included age, sex, weight, body mass index (BMI), ethnicity, ICU service, vasoplegia etiology, baseline NEE requirements, B12 infusion time, B12 dose, mechanical ventilation, incidence of fluid administration within 12 hours before and after B12, type of fluid administered, and volume of fluid administered. Vasoplegia’s etiology was determined by review of provider notes within the patients’ chart.

Statistical analysis

Descriptive statistics were utilized for all outcomes and demographic data. The primary outcome, duration of response, is reported as median and range. Patient characteristics and secondary outcomes are summarized by number and percentage (%) or median and interquartile range (IQR). Fluid administration data is summarized by number and percentage (%) or mean and range. 

Results from this study were previously presented as a virtual abstract at the Society of Critical Care Medicine's 2022 Annual Critical Care Congress in February 2022 [24].

## Results

Study population 

A total of 16 patients received IV hydroxocobalamin between June 2018 and June 2021 at this institution. All 16 patients were included in this study. Five patients were deemed “responders” and 11 patients were “non-responders”. Patient demographics and clinical characteristics are described in Table 1. Most patients resided in the cardiovascular ICU when they received B12 (87.5%), and the most common vasoplegia etiology was sepsis (68.8%). Responders had baseline NEE requirements > 30mcg/min, were mechanically ventilated, received 5 grams of IV B12, and only one patient received an extended infusion (over six hours). Fluid administration within 12 hours before and after B12 administration is described in Table 2 for both responders and non-responders.

**Table 1:** Characteristics Variables are expressed as median (IQR) and n (%).

Characteristic	Responders (n=5)	Non-responders (n=11)	Total (n=16)
Age, years	54 (36-70)	62 (46-67)	61 (38.5-67)
Male	3 (60)	10 (91)	13 (81.3)
Weight, kg	83.1 (81.3-105.2)	100.5 (90-107.7)	95 (82.4-106.8)
BMI, kg/m2	28.6 (26.9-34)	30.9 (27.5-33.1)	29.6 (27.1-33.1)
Ethnicity			
White	5 (100)	8 (73)	13 (81.3)
Black or African American	0 (0)	1 (9)	1 (6.3)
Unknown/Not Reported	0 (0)	2 (18)	2 (12.5)
ICU service			
Cardiovascular	4 (80)	10 (90.9)	14 (87.5)
Thoracic	1 (20)	0 (0)	1 (6.3)
Medical	0 (0)	1 (9.1)	1 (6.3)
Shock etiology			
Sepsis	3 (60)	8 (72.7)	11 (68.8)
Cardiopulmonary bypass	2 (40)	2 (18)	4 (25)
Acute liver failure	0 (0)	1 (9.1)	1 (6.3)
Baseline NEE requirements, mcg/min	33 (19.2-34.1)	24.7 (22.2-27.3)	25.4 (19.8-33.8)
Mechanical ventilation	5 (100)	10 (90.9)	15 (93.8)
Hydroxocobalamin dose			
5 grams	5 (100)	10 (90.9)	15 (93.7)
10 grams	0 (0)	1 (9.1)	1 (6.3)
Hydroxocobalamin infusion time			
5 g over 15 minutes	4 (80)	10 (90.9)	14 (87.5)
5 g over 360 minutes	1 (20)	1 (9.1)	2 (12.5)

**Table 2:** Fluid characteristics Variables are expressed as mean (range) and n (%).

Characteristic	Responders (n=5)	Non-responders (n=11)
Received intravenous fluids within 12 hr before hydroxocobalamin	1 (20)	9 (81.8)
Volume administered		
Albumin 25%, mL	0, n=0	100, n=1
Albumin 5%, mL	0, n=0	650 (250-1250), n=5
Lactated Ringers, mL	0, n=0	500, n=1
Normal Saline, mL	1500, n=1	437.5 (250-500), n=4
Received intravenous fluids within 12 hr after hydroxocobalamin	3 (60)	7 (63.6)
Volume administered		
Albumin 25%, mL	0, n=0	100, n=2
Albumin 5%, mL	250, n=1	650 (500-1000), n=5
Lactated Ringers, mL	0, n=0	2250 (500-4000), n=2
Normal Saline, mL	375 (250-500), n=2	1125 (1000-1250), n=2

Outcomes 

**Figure 1:**
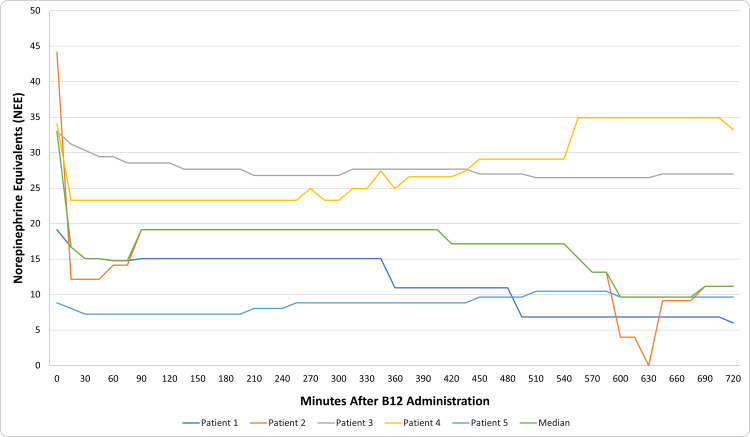
'Responder' vasopressor requirements over time

The primary outcome of IV B12 effect duration in responders was 210 minutes. All recorded vasopressor requirements during the 12-hour observation period for each responder are displayed in Figure 1. The time to respond was rapid (median 15 minutes), and the range of effect duration was 60 - 315 minutes. Median NEE requirements decreased from 32.9 mcg/min at baseline to 16.7 mcg/min at the first 15-minute time point and 14.8 mcg/min at one hour after B12 administration. At hour 12, 3 (60%) patients required less NEE than before B12 administration, despite requiring an increase during the first five hours. 

Secondary outcomes for both responders and non-responders are displayed in Table 3. Hospital and ICU LOS for the cohort were 18.6 days and 17.7 days, respectively. In-hospital mortality was common and occurred in almost 90% of all patients. 

**Table 3:** Secondary outcomes Variables are expressed as median (IQR) and n (%). ICU = intensive care unit; LOS = length of stay.

Outcome	Responders (n=5)	Non-responders (n=11)	Total (n=16)
Hospital LOS, days	27.5 (24.8-51.2)	17.2 (12.9-25.3)	18.6 (14.8-38.2)
ICU LOS, days	27.5 (11.7-46.6)	12.6 (4.2-18.4)	17.7 (4.9-37)
In-hospital mortality	4 (80)	10 (90.9)	14 (87.5)

## Discussion

This was the first study performed to quantify the duration of the effect on vasopressor requirements in patients that respond to IV B12 in the Intensive Care Unit (ICU). The 210-minute duration of effect observed in this study suggests the maximum benefit of B12 is short-lived, but there may be some persistent, low-level effects as 60% of responders required less NEE 12 hours after administration compared to baseline.

Our results are consistent with those of Armour and colleagues, as they reported hemodynamic values returning to baseline 210 minutes after B12 administration [8]. It was noted, however, norepinephrine and vasopressin requirements remained lower than baseline for the duration of the 240-minute observation period [8].​ These evaluations included all patients, regardless of “responder” status, and explicitly excluded those that did not receive cardiopulmonary bypass. 

Our results are also consistent with those of a more recent study by Patel and colleagues, which reported the feasibility of a placebo-controlled trial with IV B12 [25]. This study reported NEE reductions of approximately 40% at 30 minutes post-IV B12 infusion and sustained reductions at three hours post-infusion [25]. This study also examined changes in hydrogen sulfide levels after IV B12 administration, introducing a new monitoring parameter not previously utilized in other literature [25]. This study, however, only enrolled patients with septic shock as their primary cause of vasoplegia and did not report hemodynamic results beyond three hours [25]. The current study accepted all patients regardless of shock etiology, evaluated the duration of effect for responders specifically, and utilized an observation window that was detailed (every 15-minute intervals) and extended compared to previous literature. 

Response to B12 was low (31%) within our cohort, and in-hospital mortality was very high (87.5%). Two other reports have attempted to delineate responders vs. non-responders and found a 47% and 65% response rate in cohorts of 17 and 66 post-cardiopulmonary bypass patients, respectively, which is a higher response rate than we observed in our mixed cohort [14,18]. This is likely due to the definitions used for “responders”. Shah and colleagues created trajectory models and categorized them based on visual inspection [14]. Vollmer and colleagues utilized a definition of any reduction in vasopressor requirements two hours after B12 administration, which may be subject to natural variations in blood pressure [18]. The mortality incidence observed in our study is similar to previous studies, as published reports from DePrima, Sacco, and Seelhammer observed mortality rates of 78%, 73%, and 50%, respectively [3,7,10]. 

This study has several strengths. Responders were delineated from non-responders using objective criteria to obtain effect duration only from those that received a hemodynamic benefit from B12. The 10% reduction of vasopressors within one hour was selected in part due to the findings of DePrima, who noted a 10% decrease in NEE from baseline one hour after B12 administration [10]. This study also included any etiology of shock and was able to provide a quantification of responders, which has only been attempted in two other studies [14,18]. 

This study had several limitations. Due to the limited use of B12 as a treatment for catecholamine refractory shock, our sample size was small but comparable with previous reports [3,7,8,10-12,14,17,18]. We were not able to control for other interventions that were performed that may have impacted vasopressor requirements due to the retrospective design, but we did report variables that were available based on existing documentation. The etiology of vasoplegia was based on a review of provider notes, which could be subject to omission or conjecture.

## Conclusions

The overall response rate to IV B12 was low, and mortality was high in this cohort. For those who responded to IV B12, the duration of effect was 210 minutes. This therapy is reasonable to consider in cases of vasoplegia, but a short duration of effect and low response rates should be expected.
